# Red Water

**Published:** 1901-11

**Authors:** W. H. Dalrymple

**Affiliations:** State Experiment Station, Baton Rouge, La.


### "RED WATER."

EDITORS JOURNAL OF COMPARATIVE MEDICINE, ETC.:

IN the section of the September issue of the JOURNAL devoted to "Reports of Cases," Dr. J. M. Gibb, Government Inspector of Stock, Kingston, Jamaica, gives the history and therapeutic treatment of a bovine malady which he terms "red water," and which appears to be of a very fatal character among the imported cattle on that island. After a careful perusal of the history, the symptoms, and the thermometric records so carefully obtained by Dr. Gibb, and from the experience I have had with our "Southern cattle fever," I am very much inclined to the opinion that he is confronted with a condition closely akin to that disease. In fact, I am very much impressed with the idea that the so-called "red water" of our European authors is also of protozoönic origin, for I have observed in a somewhat recent issue of the London *Veterinary Record* that Professor Mettam, of the Royal Veterinary College of Ireland, found the "pyrosoma bigeminum" in the blood of a "red water" victim in the neighborhood of Dublin.

As to whether the "boophilus bovis" exists on the Island of Jamaica I am not aware, but there may be other media through which the protozoön may be transmitted from native or immune cattle to newly imported susceptible ones. If Dr. Gibb could identify the organism by an examination of the blood, that would settle the question as to the character of the disease, then I would unhesitatingly suggest artificial immunization of all imported cattle by blood-inoculation, which has given such splendid results in the case of our Southern cattle fever. After reading Dr. Gibb's report of his cases in the JOURNAL, I had forwarded to him copies of our Louisiana Experiment Station Bulletins bearing upon the subject of Texas fever and the immunization treatment, and which, I trust, may prove useful to him in the event of the "red water" with which he has had to deal being a somewhat close relative to the malady which heretofore has been the bugbear of our Southern importers of blooded cattle from above the Federal quarantine line.

W. H. DALRYMPLE, M.R.C.V.S.

STATE EXPERIMENT STATION, BATON ROUGE, LA.

